# The Use of Natural Rubber as an Initiator of LDPE Biodegradation in Soil

**DOI:** 10.3390/polym17212885

**Published:** 2025-10-29

**Authors:** Ivetta Varyan, Polina Tyubaeva, Matheus Poletto, Egor S. Morokov, Anastasia V. Bolshakova, Svetlana G. Karpova, Evgeny A. Kolesnikov, Anatoly Popov

**Affiliations:** 1 Department of Biological and Chemical Physics of Polymers, Emanuel Institute of Biochemical Physics, Russian Academy of Sciences, 4 Kosygina Street, 119334 Moscow, Russia; polina-tyubaeva@yandex.ru (P.T.); es_morokov@yahoo.com (E.S.M.); karpova@sky.chph.ras.ru (S.G.K.); anatoly.popov@mail.ru (A.P.); 2 Center for Collective Use «Scientific Equipment», Plekhanov Russian University of Economics, 36 Stremyanny Lane, 117997 Moscow, Russia; 3 Postgraduate Program in Engineering of Processes and Technologies, University of Caxias do Sul, Caxias do Sul 95070-560, Brazil; mpolett1@ucs.br; 4 Chemistry Department, Moscow State University, Lenlinskie Gory 1 Str 3, 119991 Moscow, Russia; nastya@polly.phys.msu.ru; 5 Department of Functional Nanosystems and High-Temperature Materials, National University of Science and Technology (MISIS), Leninsky Prospekt, 4-1, 119049 Moscow, Russia; kolesnikov.ea@misis.ru

**Keywords:** biodegradation, polyethylene, natural rubber

## Abstract

The control of the quantities of multi-tonnage polymers, in particular, making them biodegradable, is an urgent task. This study suggests a new approach in the application of natural rubber (NR) as an initiator of biodegradation of low-density polyethylene (LDPE) in soil. The study examines the structure, properties and rates of biodegradation of thin LDPE films with different content of NR. Such methods as fourier transform infrared spectroscopy (FTIR), electron paramagnetic resonance (EPR), differential scanning calorimetry (DSC), thermogravimetric analysis (TGA), scanning electron microscope (SEM), atomic force microscopy (AFM), gel-permeation chromatography (GPC), and acoustic microscopy were used for the most complete characterization of NR/LDPE composite systems. It was shown for the first time that at concentrations above 30%, NR is able to form an interpenetrating structure with the LDPE matrix, which has a decisive effect on the initiation of biodegradation during exposure in soil. Thus, the composition with 50% natural rubber exhibits the highest mass loss. The sample with 50% natural rubber content lost 70% of its mass, while the one with 40% NR content lost 38%. Furthermore, after soil burial, a significant decrease in crystallinity was observed: from 39.5% to 31.5% for the 90/10 composition and from 39.1% to 24.2% for the 50/50 composition. The results obtained are confirmed by a noticeable decrease in the molecular weight characteristics of LDPE.

## 1. Introduction

One of the most pressing environmental problems of our time is the global accumulation of plastic waste, a significant portion of which is disposable packaging made from polyolefins, primarily low-density polyethylene (LDPE). According to numerous studies, up to 12 million tons of plastic waste enters the global environment each year, a significant portion of which is packaging materials [1,2,3,4,5,6,7]. The resistance of polyethylene to microorganisms, photodegradation, and moisture is due to its chemical inertness and the presence of ordered crystalline regions, which significantly impedes its natural decomposition processes [3,8,9].

Thin polyethylene films used in packaging, agriculture, and household applications are of particular concern. Their thinness and high specific surface area accelerate fragmentation, but their resistance to biodegradation leads to the accumulation of microplastics—products that can enter the food chain [10,11].

With the tightening of environmental standards and the emergence of international initiatives aimed at reducing plastic pollution (including EU directives and UNEP initiatives), there is a need to develop new polymer materials that combine the properties of traditional polyolefins with the possibility of controlled degradation under natural conditions [12,13,14].

Existing approaches to controlling the kinetics of polyethylene and other polyolefin degradation can be divided into three main groups: the introduction of pro-oxidants and oxidation initiators, compounding with biodegradable polymers and natural fillers, and chemical functionalization of the base polymer [15,16,17]. The most technologically advanced method is the addition of pro-oxidants, including organometallic compounds (Fe, Mn, Co) or organic peroxides, which stimulate thermal and photo-oxidative degradation of polyethylene with the formation of carbonyl and hydroxyl groups that can be further attacked by microorganisms [18,19]. However, there is ample evidence that such materials do not demonstrate complete biodegradability under natural conditions and often break down only into small fragments—microplastics [20,21,22].

Another promising direction is the physical blending of polyolefins with biodegradable components, both synthetic (PLA, PCL, PHB) and natural (starch, lignin, cellulose, natural rubber). Such composites potentially combine the properties of the original components, and the biodegradable phase can act as an “entry point” for microbiota, facilitating access to the polyolefin matrix [23]. The structure of the interphase boundary plays a special role here: the presence of a continuous biodegradable phase, a developed interface, and hydrophilicity significantly affect the efficiency of destruction [24,25].

Along with physical mixing, another approach is being developed: chemical modification of polyolefins. The most common techniques are grafting of maleic anhydride and other polar functional groups, as well as the use of reactive copolymers and surface activators. Such additives act as compatibilizers, improving surface wettability, adhesion, and biofouling, which is confirmed by data on increased interphase interaction in polyolefin compositions [23,26,27,28].

Rubber–plastic blends represent a class of thermoplastic elastomers widely used in the production of various technical and consumer goods. The key characteristic of these blends is the two-phase nature of the system. One phase constitutes a rigid polymer domain that does not melt at room temperature but begins to melt at elevated temperatures, and the other phase is the soft rubber phase [29]. Such blends possess many properties of rubbers and, at the same time, can be processed like thermoplastics. The majority of scientific studies are devoted to investigating blends of natural rubber with commodity polyolefins, polyethylene and polypropylene. Blending natural rubber with a polyolefin yields thermoplastic natural rubber (TPNR). The properties of such a blend range from soft to semi-rigid materials. In the scientific literature, the results of studies on the properties of natural rubber/plastic blends are most extensively presented by authors from countries producing natural rubber, primarily in Southeast Asia. An additional advantage stimulating research in these countries is the low cost of the produced materials. The main research focus is on the physico-mechanical properties of the blends and improving component compatibility. The properties of thermoplastic elastomers can be easily tailored by changing the blend composition. In [30], the authors studied the effect of LDPE/NR blend composition on the failure mechanism (crack formation) using scanning electron microscopy. The PE content in the blends varied from 100 to 30 wt.%. The influence of rubber domain sizes and the transition to an interpenetrating two-phase system on blend failure was tracked. However, the problem of technological compatibility in thermoplastic natural rubbers still persists and receives significant attention. Such deformation-strength properties as elastic modulus and strength depend on the properties of the blend components, phase morphology, and adhesion between the components. The interaction between components plays a dominant role in determining the performance properties of the materials [31].

Thus, the creation of biodegradable polyethylene materials requires not only the selection of a suitable modifier, but also an understanding of the structural and property relationships that determine the mechanism of biodegradation: from morphology and hydrophilicity to mechanical properties and soil stability [32,33,34]. Among the many possible solutions, natural rubber has attracted the most attention in recent years as a biodegradable, technologically compatible, and structurally active component of low-density polyethylene-based materials [14,22]. Therefore, studying the effect of NR additives on the biodegradability of LDPE-based blends under the influence of soil microorganisms is of scientific and practical interest. Moreover, the physical and mechanical properties of the resulting materials must meet the required performance characteristics.

Due to the combination of several positive qualities of this material (low cost and ease of manufacture, good biodegradability, and acceptable mechanical properties), we conclude that further research into composites of this type is extremely important.

Based on the foregoing, the aim of this work was formulated: to establish the nature of the influence of the composition and structure of low-density polyethylene (LDPE) and natural rubber (NR)-based blends on their properties and biodegradability. Specifically: development of a technology and formulation for producing biodegradable composite materials based on low-density polyethylene and natural rubber with required performance properties, including strength; identification of the patterns of natural rubber’s influence on the physical, chemical, and mechanical properties of blends of varying composition; investigation of the ability of the developed materials to undergo biodestruction under the influence of various physical, chemical, and biochemical factors under laboratory conditions; analysis of the biodegradation process using various physico-chemical methods, as well as by changes in the molecular weight and molecular weight distribution of LDPE.

## 2. Materials and Methods

### 2.1. Materials

This research examined compositions formulated from low-density polyethylene (LDPE, grade 15803-020, OJSC Neftekhimsavilen, Moscow, Russia) incorporating natural rubber (NR, grade SVR-3L, Ho Chi Minh, Vietnam) as an additive. The characteristics that describe the poly-mer chain structure of the low-density polyethylene are presented in Table 1.

Natural rubber was used as a natural biodegradable additive. Natural rubber is a biopolymer consisting of 91–96% stereoregular cis-polyisoprene. Due to the regular arrangement of isopentenyl units in the 1,4-cis position, natural rubber has low brittleness and glass transition temperatures (around −70 °C). The natural rubber used in this work consists of poly(cis-1,4-isoprene) with insignificant impurities of other compounds and water. The composition of natural rubber is shown in Table 2.

**Figure 1:**
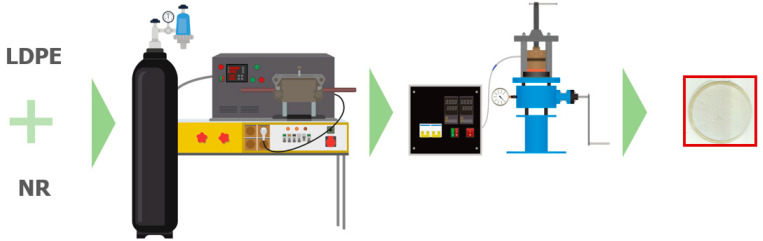
The process of obtaining film samples.

The general process of obtaining film samples is shown in the diagram (Figure 1).

LDPE compositions with NR were prepared using a Brabender laboratory rotary mixer (ICP, Moscow, Russia). The total weight of the sample was 20 g. The mixing process was carried out at a temperature of (140 ± 2) °C in an argon atmosphere. At a rotor speed of 15 rpm, the crushed natural rubber was loaded into the mixing chamber. After 1 min, the rotor speed was increased first to 30 rpm and then to 60 rpm. Then, at the 7th minute, polyethylene granules were added, the roller speed remained at 60 rpm, and mixing continued for 7 min. The total preparation time of the composition was 14 min. Following the mixing process, the compound was extracted from the mixing chamber and allowed to cool to ambient temperature. The concentration of natural rubber in the composites was set at 0, 10, 30, and 50 percent by weight. Film samples were produced from the comminuted mixtures.

The shredded mixtures were compression-molded using a laboratory hydraulic press featuring electronically heated plates. The process was conducted at a temperature of 140 °C and a pressure of 40 kgf/cm^2^, utilizing a cellophane substrate. The samples of the shredded mixture were spread evenly across the surface of the substrate.

The pressing time was 2 min. The resulting samples were then quenched in water at (20 ± 2) °C. This resulted in round film samples with a diameter of 7 cm and a thickness of (160 ± 10) μm.

### 2.2. Methods

#### 2.2.1. Optical Microscopy

The study of the material structure, along with the assessment of the uniformity of the natural rubber phase distribution within the polyethylene matrix, which includes the influence of the dispersed filler’s scale factor, was performed using an optical microscope from Axio Imager Z2m (Carl Zeiss, Oberkochen, Germany). This analysis utilized the Axio Vision software Viewer 4.8 at magnifications of 50× and 200× in both transmitted and reflected light modes. The images were recorded as microphotographs.

#### 2.2.2. Scanning Electron Microscopy

Scanning electron microscopy (SEM) was performed using a Tescan VEGA3 scanning electron microscope (Tescan, Czech Republic) with a platinum conductive coating on all samples. The accelerating voltage was 20 kV. The scanning electron images were analyzed using Femto Scan Online software.

#### 2.2.3. Atomic Force Microscopy

Atomic force microscopy measurements were performed on NanoScope V Multimode (Bruker, Bilerica, MA, USA) operating in tapping mode with Tips Nano polysilicon cantilevers (resonant frequency 77 kHz, Q-factor 300, Watsonville, CA, USA). All images were prepared by the Femto Scan Online software (Advanced Technologies Center, Russia, version 2.3.220). Average roughness (R_a_) and average square roughness (R_q_) were measured according to the standard technique using the Femto Scan Online software [35]. The measurement was carried out along the scanning direction along five sections.

#### 2.2.4. Ultrasonic Microscopy

Ultrasonic studies of the internal microstructure of polyethylene-based composite films with different concentrations of natural rubber were conducted using the SIAM-2017 acoustic microscope, developed and designed in the acoustic microscopy laboratory of the IBCP RAS [36]. The acoustic lens was operated at a working frequency of 100 MHz and an angular aperture (half the angle of opening) of 11°. The acoustic lens was fixed in a precision mechanical scanning system (XYZ axis) with a scanning step of 5 μm (the reproducibility of the lens position is 0.5 μm). When scanning a sample with a lens, the ultrasonic beam passes through immersion (water-based acoustic gel), reflects from the surface and bottom of the sample, elements of the internal structure, and is received by the same acoustic lens. The received signals are digitized by a 12-bit ADC with a sampling frequency of 1 GHz (2 × 500 MHz) and averaged 16 times at each observation point. The resulting echo signal includes time-resolved echo signals originating from elements or boundaries located at different depths within the sample volume.

#### 2.2.5. Biodegradation

To study the biodegradability of materials, an experiment was conducted in laboratory conditions using specially prepared soil that mimics the natural soil environment. The soil mixture was prepared in accordance with the requirements of GOST 9.060 “Unified system for protection against corrosion and aging. Fabrics. Laboratory test method for resistance to microbiological degradation”. The soil components were garden soil, sand, and horse manure, mixed in equal proportions by mass.

During the tests, the key parameter was soil moisture, which was maintained at an optimal level for microbial activity—60 ± 5%. This parameter was regularly monitored using an ETP-301 soil moisture meter, and periodic watering was performed to stabilize it.

**Figure 2:**
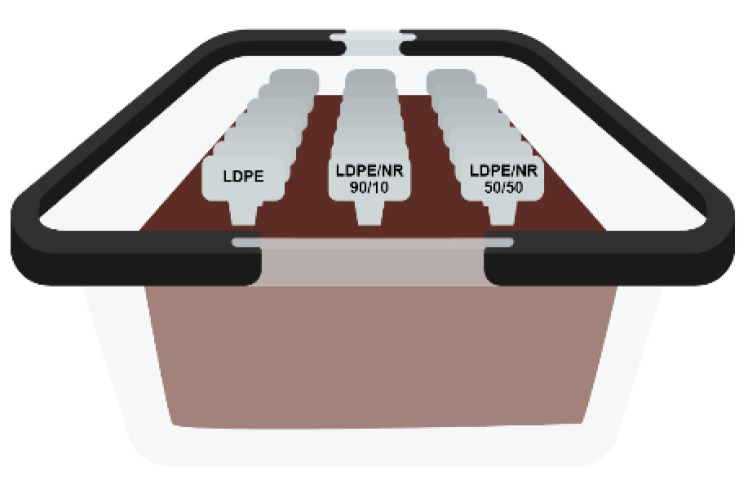
Photo of experimental equipment for samples exposure in soil.

The experiment was conducted in containers measuring 40 × 50 × 35 cm, which were placed in a room with a constant air temperature of 22 ± 2 °C. The film samples were placed vertically in the soil, located in the central part of the volume, where they were kept for specified periods of time: 6, 12, 17, 24, 31, 42, and 60 months (Figure 2).

Next, at each time interval, samples were extracted from the soil, cleansed of soil residues, and air-dried until a constant mass was achieved. Subsequently, a visual inspection was conducted to evaluate changes in color and any loss of transparency in the composites, analysis of changes in sample mass, changes in chemical composition, and assessment of changes in structure, bacterial growth, and biofouling of the samples.

The visual assessment was carried out by comparing the color change of the samples and the loss of transparency. Also, the change in the mass of the samples was considered.

#### 2.2.6. Differential Scanning Calorimetry

Differential scanning calorimeter Netzsch 214 Polyma (Netzsch, Selb, Germany) was used for evaluation the of thermophysical properties of LDPE and LDPE/NR samples. All measurements were carried in the nitrogen atmosphere according to the standard technique [37]. Heating and cooling rate was 10 °K/min. Test samples were cut from 3 different areas from the film with total weight of 7 mg. DSC curves were analyzed using Netzsch Proteus software. The peak temperature of melting and enthalpy of melting were determined according to the technique [37]. The degree of crystallinity for each sample was determined from its melting enthalpy, applying a correction for the natural rubber content present in the sample, using the formula:

Χ_*LDPE*_ = Δ*H_LDPE_*/Δ*H*_100%*PE*_ × *C_LDPE_* × 100% (1)

where: *Χ_LDPE_*—the crystallinity degree of LDPE; Δ*H_LDPE_*—the enthalpy of melting of LDPE determined by the DSC; Δ*H*_100%*PE*_—the enthalpy of melting of the 100% crystalline PE, which was 280 J/g [38]; *C_LDPE_*—the content of the LDPE in the composition.

#### 2.2.7. Mechanical Analysis After Exposure to Soil

The physical and mechanical properties of the materials following a five-year soil burial test were evaluated on a GPUG5 DLC-0.5 DVT Devotrans (Istanbul, Turkey) universal tensile testing machine from Istanbul, Turkey, in compliance with the GOST 34370-2017 standard. Specimens were die-cut from the films with a 40 mm gauge length and tested at a cross-head speed of 100 mm/min. For every composite formulation, seven replicate tests were conducted.

#### 2.2.8. Thermogravimetric Analysis

In order to evaluate the effect of temperature on the mechanism of thermal destruction of samples, a study was conducted using thermogravimetric analysis (TGA) with a Mettler Toledo TGA/DSC3+ (Greifensee, Switzerland) under dynamic heating conditions at a heating rate of 10 °C/min in a nitrogen atmosphere of 20 mL/min. The sample weight was 4–5 mg.

#### 2.2.9. Electron Paramagnetic Resonance

In this work, molecular dynamics studies were performed on an automated EPR-V spectrometer (Federal Research Center of Chemical Physics, Russian Academy of Sciences, Moscow). X-band electron paramagnetic resonance (EPR) spectra were recorded and calculated, obtaining data on the molecular dynamics of polymer samples. The microwave power was maintained at or below 1 mW to prevent saturation effects. The amplitude of modulation was kept lower than 0.5 G, ensuring it was always less than the resonance linewidth. The stable nitroxide radical TEMPO served as the spin probe. Introduction of the radical into the fibers was achieved from the gas phase over a 30-min period at 50 °C. The radical concentration within the polymer was ascertained by integrating the EPR spectra. An evacuated TEMPO solution in carbon tetrachloride (CCl_4_) with a radical concentration of approximately 1 × 10^−3^ mol/L was used as the standard. The equilibrium concentration of the adsorbed radical in samples of equal mass was calculated using Brucker (Winer) software. During spectral recording, the gain was recorded, the sample was weighed, and then the radical concentration in each sample was calculated using Origin software.

To calculate the correlation time of rotation of nitroxyl radicals in the range of 5 × 10^−11^ < τ <1 × 10^−9^ from the experimental spectra, the following relation was used:

τ = Δ*H*_+_ × [(I_+_/I_−_)^0.5^ − 1] × 6.65 × 10^−10^ (2)

where: Δ*H*_+_ is the width of the spectrum component located in the weak field, I_+_/I_−_ is the ratio of the intensities of the components in the weak and strong fields. The measurement error of τ was ±5%.

#### 2.2.10. Fourier Transform Infrared Spectroscopy

Changes in the chemical composition of the samples were determined by IR spectroscopy using a Perkin Elmer Spectrum 100 FTIR spectrometer (Rodgau, Germany) at a temperature of (22 ± 2) °C in transmission mode within the range of 4000–450 cm^−1^.

#### 2.2.11. Gel-Permeation Chromatography

A sample weighing of about 15–18 mg (precise weighing) was dissolved in 8 mL of trichlorobenzene at a temperature of 160 °C for 2 h. After dissolution, the hot solution was filtered using the filtration system included in the Agilent PL SP-260 VS (Agilent Technologies, Santa Clara, CA, USA) sample preparation station into a 2 mL vial for analysis.

The polymer samples were analyzed on an Agilent PL-GPC 220 instrument using three Agilent PLGel Olexis 300 × 7.5 mL columns connected in series.

## 3. Results and Discussion

### 3.1. Study of the Structure of the Initial Samples

Investigating the distribution of rubber particles within the LDPE matrix was a key objective. To assess the homogeneity of the natural rubber phase dispersion in the poly-ethylene matrix, the sample surfaces were analyzed via optical microscopy, scanning electron microscopy, and ultrasonic acoustic microscopy.

#### 3.1.1. Microscopy

**Figure 3:**
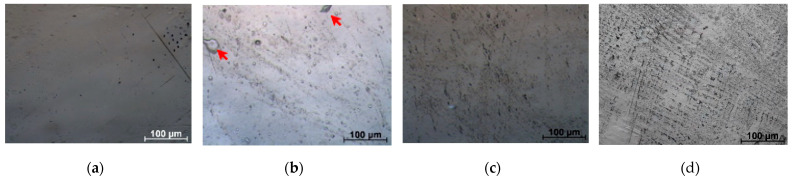
Micrographs of the original samples PE 100 (**a**), PE/NR 90/10 (**b**), PE/NR 70/30 (**c**), PE/NR 50/50 (**d**), ×200 magnification (red arrows indicate NR agglomerates).

Figure 3 shows micrographs of the surface of the initial samples.

The research established that natural rubber exhibits specific distribution characteristics within the LDPE structure. At low concentrations, it forms large particles in the polyethylene matrix, irrespective of the mixing quality and duration. With an increase in NR concentration, the average size of such inclusions decreases, reaching a minimum at an LDPE/NR concentration of 50/50. A characteristic change in the surface microrelief and a higher degree of its development with increasing NR concentration should also be noted.

At an NR concentration of 10%, the inclusion size ranges from 6 to 48 µm, with the most frequent inclusions being 10–14 µm; most inclusions have a spherical or rounded shape. With an increase in NR concentration to 30%, the maximum size of rounded inclusions decreases to 15 µm, and elongated, thin inclusions resembling veins, with lengths up to 18–24 µm and widths up to 5–7 µm, appear. When the NR concentration reaches 50%, the shape and structure of the inclusions become difficult to identify, as a complex pattern of interconnected veins appears. Some elements of this network structure reach 40–44 µm in length and 5–7 µm in width, with many of them connected crosswise and having thickenings at the ends, resembling interconnected rounded rubber inclusions sized 7–14 µm. These changes should be attributed to the formation of an interpenetrating LDPE and NR system of the “network-in-network” type at NR contents above 30%. Similar phenomena have been observed in polyurethane and polystyrene matrices upon the introduction of NR [39,40].

#### 3.1.2. Ultrasonic Studies

**Figure 4:**
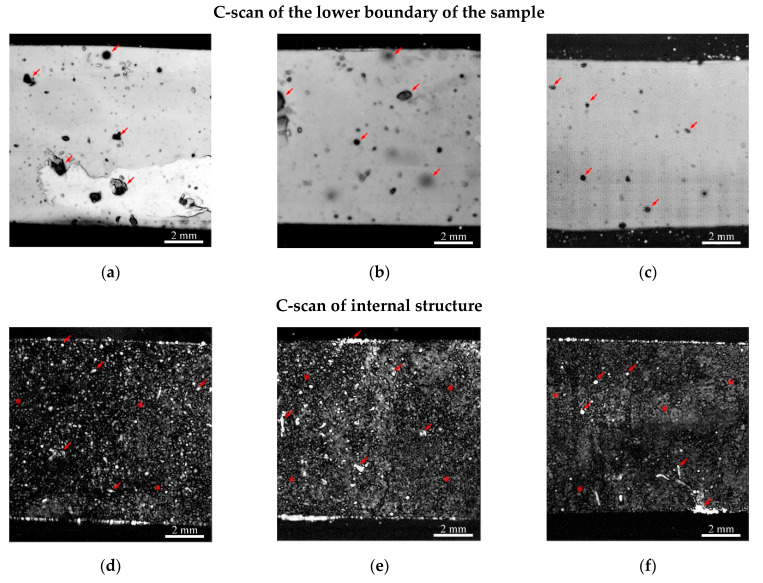
Ultrasonic images of the lower boundary of the initial samples: LDPE/NR 90/10 (**a**), LDPE/NR 70/30 (**b**), LDPE/NR 50/50 (**c**); internal structure of samples: LDPE/NR 90/10 (**d**), LDPE/NR 70/30 (**e**), LDPE/NR 50/50 (**f**). The bright and dark areas marked with arrows represent internal air inclusions, both with and without filler aggregates. The asterisks represent areas of uniform filler distribution, which small particles are represented by gray highlights.

Ultrasonic studies of the internal microstructure of polyethylene-based composite films with different concentrations of natural rubber showed that, regardless of the presence of filler, microscopic air cavities (bubbles) are present in the volume of the samples. The results are shown in Figure 4. Extended air cavities seen as dark acoustic shadows in C-scans of lower boundary of the samples (Figure 4a–c).

**Figure 5:**
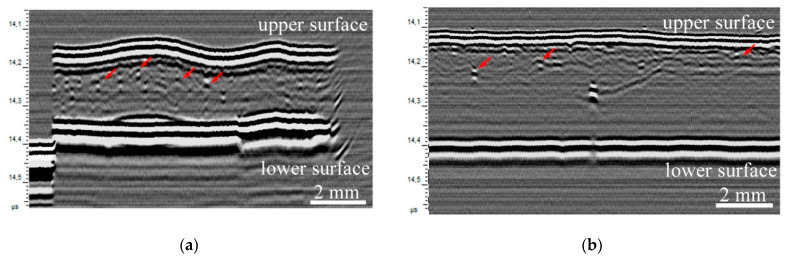
Ultrasonic vertical images of the original samples: LDPE/NR 90/10 (**a**), LDPE/NR 50/50 (**b**). Arrows are filler agglomerates.

In the volume of samples with the minimum rubber concentration, the LDPE/NR 90/10 samples (Figure 4a,d) clearly show separate particles and filler conglomerates. Due to their low concentration, they are clearly distinguishable against the background of the polymer matrix. In addition, some of the conglomerates contain air, which also affects the clear display of particles in the volume [41]. With an increase in the concentration of rubber in polyethylene, several physical effects are observed. First, there is a decrease in the amplitude of the echo signal reflected from the upper boundary due to an increase in the number of rubber particles on the surface of the samples, which have a lower acoustic impedance (reference: Acoustic impedance is the product of density and sound velocity in a material; the reflection and transmission coefficients of ultrasound at boundaries are calculated from the impedance ratios of contacting media) (Figure 5). Thus, further in the work, attention is focused on two examples of different structures: “agglomerates in a matrix” (90/10) and “network-in-network” (50/50).

Secondly, there is a decrease in longitudinal propagation velocities with increasing NR concentration (Table 3). The values of speed of sound are material characteristics that describe elastic behavior and, in combination with its density, is used for the calculation of elastic modules. Thus decrease in sound velocity depicts the drop of elastic characteristics [42].

The uniform distribution of filler throughout the volume, with the exception of visible large rubber particles, is evidenced by the minimal variation in sound velocity values across the sample. Large rubber particles cause distortions of the lower boundary of the samples in vertical images (B-scans) and shadows in C-scans of the lower boundaries of the films. These images also contain shadows created by large air inclusions in the sample volume and bubbles suspended in the immersion medium (water-based acoustic gel) above the surface.

#### 3.1.3. SEM

**Figure 6:**
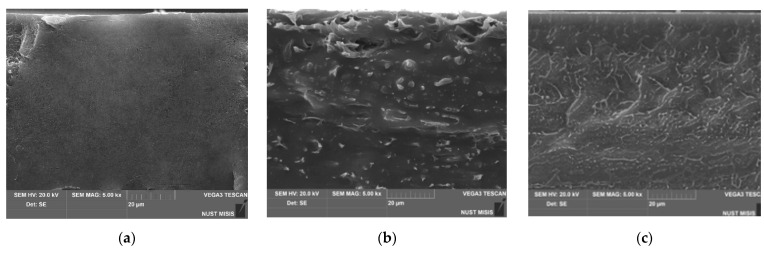
SEM photographs of LDPE/NR samples, where the component ratio was: 100/0 (**a**), 90/10 (**b**), 50/50 (**c**), respectively.

SEM images of the original films based on low-density polyethylene with the addition of natural rubber are shown in Figure 6.

A cross-section of LDPE film samples (Figure 6a) shows a uniform structure with crack-like relief in the chip area. When 10 wt.% NR is introduced, large NR inclusions become clearly visible on the chip (Figure 6b, varying in shape, including elongated oblong inclusions in cross-section. However, when the NR concentration reaches 50 wt.%, it is clearly visible that the crack-like relief of LDPE is again clearly distinguishable, while it should be noted that there are NR particles of the smallest size and highest distribution frequency. These cross-sections are fully consistent with the concept of an interpenetrating LDPE and NR system of the “mesh in mesh” type.

#### 3.1.4. Atomic Force Microscopy (AFM)

Surface roughness is an important topographical characteristic of a material’s surface, which plays a key role in the ability of microorganisms to adhere to the surface. The arithmetic mean roughness (R_a_) and the root mean square deviation of the profile height from the mean line (R_q_) are shown in Table 4 and clearly demonstrate the change in the micro-relief of the surface [43]. The introduction of NR, regardless of concentration, leads to a decrease in R_a_ and R_q_, which indicates a narrowing of the height distribution function and a smoother surface.

**Figure 7:**
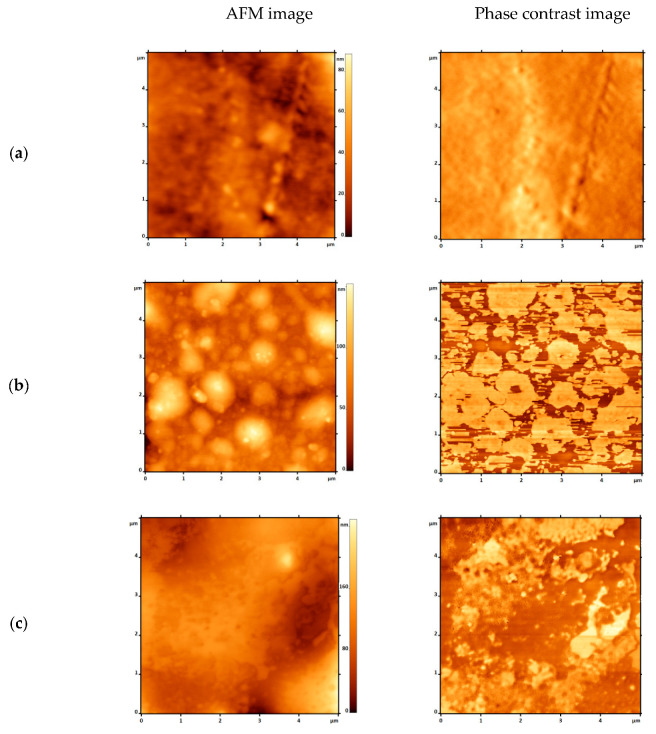
AFM images of LDPE/NR samples, where the component ratio was: 100/0 (**a**), 90/10 (**b**), 50/50 (**c**), respectively.

Of particular interest are the AFM surface images (Figure 7) and phase contrast images. Phase contrast shows that when 10% NR is introduced, the smooth, uniform surface of LDPE acquires the appearance of a structure with a clear distinction between two phases: rubber and LDPE. A bright interphase boundary is visible. NR agglomerates are visible in the AFM micrograph. In the case of 50% NR, we do not observe agglomerates, but rather an interpenetrating structure, which is consistent with the phase contrast data. As noted in Section 3.1.1, an increase in NR concentration above 30% leads to the formation of an interpenetrating structure of the network-in-network type. Given the absence of strong intermolecular interaction or chemical cross-linking between NR and LDPE, it can be assumed that a significant interphase region may be present between the composite components. It is this interphase region that is responsible for the composite’s water absorption and affects the mechanical properties of the system [44]. However, when the NR particle sizes in the composite become not only small but also quantitatively comparable to the matrix, a reorganization of this contact region between the two materials is observed. This reorganization is evident both in acoustic microscopy within the sample volume and in AFM microscopy on the surface. There is no clear boundary between LDPE and NR, which indicates the absence of a sharp interphase transition detectable by the instrument; yet, in phase contrast, the presence of two materials differing in nature (light and dark) is visible, forming an interpenetrating system [45].

### 3.2. Biodegradability Study

#### 3.2.1. Laboratory Biodegradation Study

The method of exposing material samples in soil is used to determine their biostability. Studying the degradation processes of materials in soil allows predicting their behavior during disposal by burial. To study biodegradation, a soil test was conducted using restored soil. The characteristics of material degradation were investigated by examining changes in the appearance and mass of the samples, as well as their chemical composition after exposure to soil [46,47,48].

**Figure 8:**
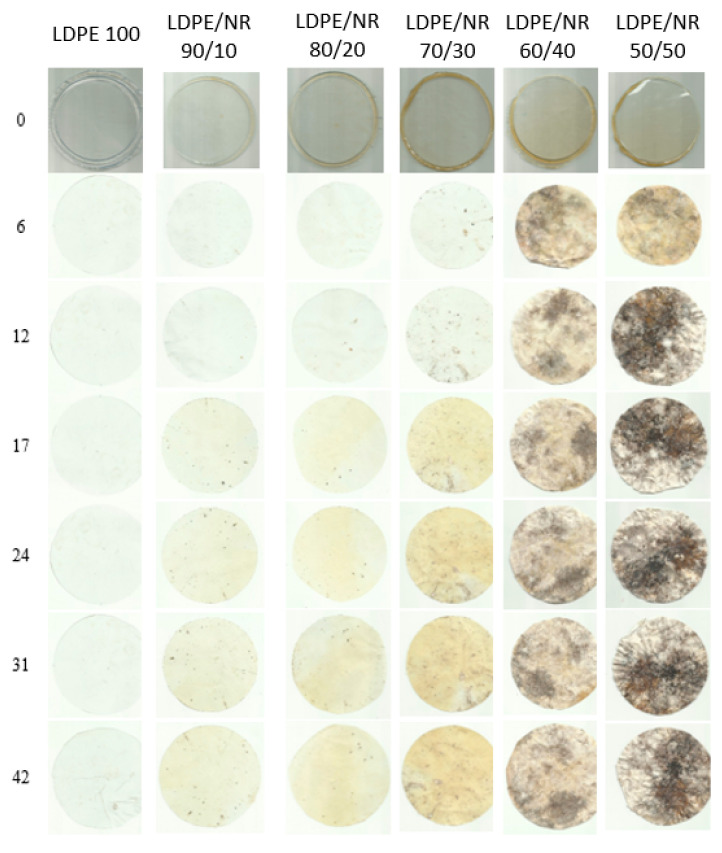
Photographs of samples of LDPE/NR compositions with NR contents of 0, 10, 20, 30, 40, and 50% before and after 6, 12, 17, 24, 31, and 42 months of exposure in laboratory soil.

A visual assessment of the appearance of PE and PE/NR composites with NR contents of 10, 20, 30, 40, and 50% showed that changes in appearance were visible on all samples; the PE/NR samples with 40 and 50% NR darkened significantly, dark inclusions and spots were observed, and small through holes appeared. With an increase in the exposure time in the soil, the number and depth of darkening increased (Figure 8). It is clearly visible that with an increase in the concentration of NR, the degree of surface erosion increases, as well as the intensity of color change.

**Figure 9:**
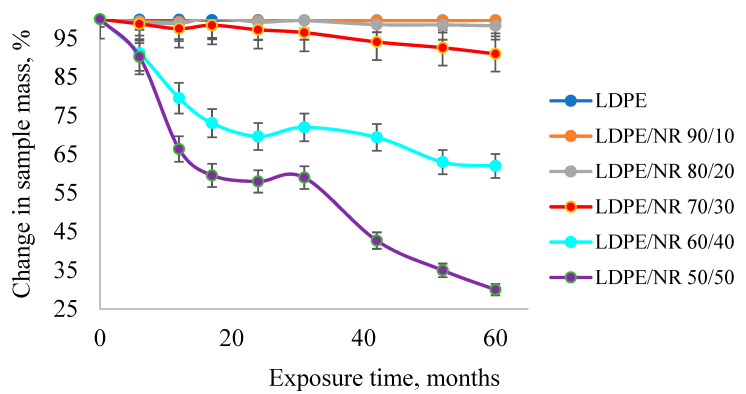
Mass loss curves for LDPE and LDPE/NR samples after exposure to laboratory soil for 0, 6, 12, 17, 24, 31, 42, 52, and 60 months.

Figure 9 shows the dynamics of mass loss of LDPE and LDPE/NR samples during 60 months of exposure in laboratory soil. A sample of pure PE was also immersed in the soil as a control sample. The mass of this control sample did not change during the entire period of exposure in the soil.

The results of mass loss measurements for the blend samples indicate that the availability of nutrients for soil microorganisms is determined by the amount of NR in the sample.

The greatest mass loss is observed for the PE/NR 50/50 blend. The graph shows that the sample with 50% natural rubber content lost 70% of its mass over 60 months, while the sample with 40% NR content lost 38%. Blends with an NR content exceeding 30% form a “network-in-network” system with a developed phase interface. At low NR content, its phase is dispersed within the PE matrix. Large NR particles are encapsulated in the polyethylene matrix, resulting in low accessibility of the rubber.

#### 3.2.2. Investigation of Properties and Changes Using FTIR Spectroscopy

To monitor the dynamics of the chemical composition of composite materials during their decomposition in soil, Fourier transform infrared (FTIR) spectroscopy in transmission mode was employed.

**Figure 10:**
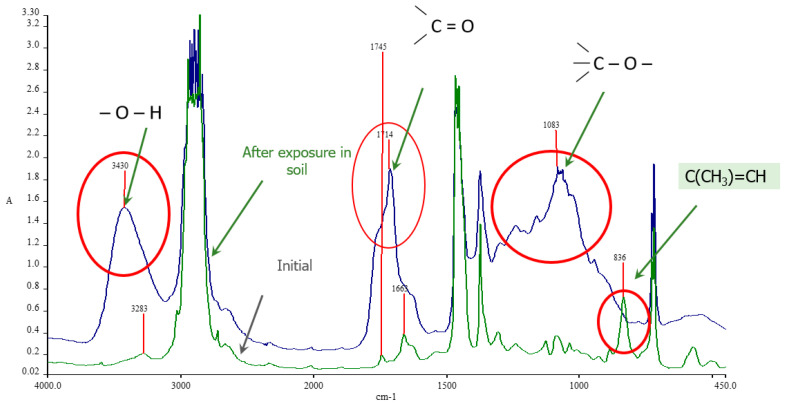
FTIR spectra of LDPE/NR 70/30 composition before (green line) and after exposure to laboratory soil for 24 months (blue line).

Figure 10 illustrates the IR spectra of the PE/NR composite (70/30) samples in their initial state (1) and after 24 months of soil exposure (2). The spectrum of the initial sample exhibited characteristic absorption bands assigned to natural rubber macromolecules. Specifically, an absorption band at 836 cm^−1^ was observed, which is associated with the C-H out-of-plane deformation vibration of the C(CH_3_)=CH group. A band at 1663 cm^−1^ was also identified, attributed to the valence vibrations of the C=C bonds in the rubber. It should be noted that the intensity of these bands increases proportionally with the NR content in the composite.

A comparison of the spectra acquired before and after biodegradation reveals that after 24 months in the soil, the bands at 836 and 1663 cm^−1^, characteristic of rubber, disappear completely. Concurrently, new broad absorption bands emerge in the IR spectrum within three regions: 3600–3000 cm^−1^, 1770–1500 cm^−1^, and 1200–900 cm^−1^. The appearance of these bands indicates the formation of various oxygen-containing functional groups in the material’s structure, such as hydroxyl (−OH) and carbonyl (C=O) groups.

#### 3.2.3. Physicomechanical Properties After Soil Exposure

The physicomechanical properties of all LDPE samples are presented in Table 5. Despite the minor changes in the crystalline phase and the compaction of the amorphous phase of LDPE, the physicomechanical parameters change significantly.

The observed reduction in the strength and deformation characteristics of the control samples also indicates physical relaxation. The probable contribution of chemical relaxation occurring during the oxidative degradation of the polymer cannot be ruled out. Although the intensity of oxidative degradation is significantly lower under shelf storage conditions, the prolonged exposure time (60 months) is accompanied by initial stages of oxidation. While no IR bands related to oxygen-containing groups are detected, the scission of macromolecules in the amorphous phase takes place and leads to its compaction. The resulting free chain fragments in the amorphous phase subsequently integrate into the crystalline structure, slightly increasing the degree of crystallinity without affecting the melting temperature.

The physicomechanical properties of all LDPE/NR 90/10 are presented in Table 6. In the control sample, which contains NR in the form of agglomerates, a similar pattern is observed as for pure LDPE. A slight decrease in all characteristics is due to the contribution of relaxation processes. At the same time, in the sample from the soil, in which NR is missing as confirmed by the FTIR and TGA (Figure S1) results, a more intense decrease in strength characteristics is observed. Thus, the intensity of the decrease in strength compared to pure LDPE increases by 18% instead of 8%.

The physicomechanical properties of all LDPE/NR 50/50 samples are presented in Table 7. The most noticeable decrease is observed in the elongation at break, which drops from 450% to 250%, while other strength parameters decrease by 15–25%. Furthermore, it should be noted that the IR spectra of the control LDPE/NR 50/50 samples reveal characteristic absorption bands of C=O, C-O, and O-H groups in the regions of 1700, 1100, and 3300 cm^−1^, respectively. Additionally, DSC (Figure S2) analysis shows a 13% increase in crystallinity for the control LDPE/NR 50/50 samples. This, together with the decrease in physicomechanical parameter values, indicates oxidative chain scission of LDPE macromolecules, i.e., a process of chemical relaxation.

It should be noted that when comparing the physicomechanical properties of the control LDPE/NR 50/50 composition and the one aged in soil, it is necessary to consider not only the absence of NR after soil exposure but also the significantly higher degree of oxidation, which is confirmed by IR spectroscopy data. Thus, it can be concluded that the biodestructive processes occurring in the samples in the soil have a significant influence. These processes are accompanied by the complete consumption of NR by the soil microbiota and concomitant enhanced oxidative degradation of LDPE.

It was found that the complete utilization of NR by microorganisms occurs within the first 24 months of exposure in the soil, according to FTIR spectroscopy results. Therefore, natural rubber is the cause of active degradation in compositions with low-density polyethylene. Natural rubber in its pure form, without natural additives, oxidizes much faster than polyethylene when exposed to oxygen. It is known that NR exhibits higher stability in the presence of additives. However, natural compounds contained in NR, including amino acids, proteins, fatty acids, carotene, metal salts, etc., are either relatively quickly consumed by microorganisms or diffuse out of the sample under soil conditions.

A large interface exists between the two incompatible components, LDPE and NR, due to the distribution of NR throughout the entire volume of the composition. The area of the interphase layer far exceeds the sample’s surface area, making the entire sample volume accessible to degrading agents (oxygen, enzymes, metabolites of microorganisms, water, etc.). The active biodestructive process of the samples in the soil leads to a significant accumulation of oxygen-containing functional groups and scission of macromolecules compared to the control samples.

The degradation of LDPE macromolecules occurs primarily in the amorphous phase but also affects the crystalline phase, a reduction of which was recorded by DSC. The accumulation of oxygen-containing groups increases the stiffness of the system due to enhanced intermolecular interactions. This leads to a sharp decrease in the deformation indicators of LDPE/NR 50/50—from 250% to 45% compared to the control, and when compared with pure LDPE aged in soil—from 580% to 45%. At the same time, strength indicators compared to the control increase, especially the elastic modulus—from 23 MPa to 81 MPa, due to intermolecular interactions, which exceeds the elastic modulus of pure LDPE (55 MPa).

Let us consider the changes in the thermal stability of samples that were in the soil for 5 years compared to the control samples.

#### 3.2.4. Investigation of Thermophysical Characteristics by DSC

The study of thermophysical characteristics by DSC allows for assessing the supramolecular structure of LDPE upon the addition of NR. It was established that the addition of less than 50% NR does not have a noticeable effect on the crystallization of LDPE or the state of its crystalline structure. The melting temperature ranges from 108 to 110 °C, and the degree of crystallinity falls within the range of 33.5 ± 1%.

When evaluating the thermophysical characteristics of the control samples by DSC, attention should be paid to the first melting event, which reflects the relaxation processes that occurred over the 5-year period. It was found that the crystallinity of pure LDPE increased by 3%, while in all LDPE/NR systems, the degree of crystallinity increased by 5–7%, although the melting temperature for all compositions remained almost unchanged, fluctuating in the range of 107–109 °C.

It is also important to note that in the LDPE/NR compositions 90/10 and 50/50 after soil exposure, no NR remains, as confirmed by IR and TGA data. The melting temperature of LDPE in the compositions did not change, remaining in the range of 108–109 °C, while the degree of crystallinity decreased noticeably from 39.5% to 31.5% for the 90/10 composition, and from 39.1% to 24.2% for the 50/50 composition. The significant decrease in the degree of crystallinity is consistent with the substantial drop in the strength characteristics of the samples after soil exposure.

#### 3.2.5. Investigation of LDPE/NR by EPR Method

According to EPR results, the amorphous phase in the control LDPE sample becomes more rigid, as the correlation time of the nitroxyl radical probe increases from 4.9 × 10^−10^ s to 5.9 × 10^−10^ s. The observed compaction of the amorphous phase, together with the DSC results, indicates physical relaxation.

Regarding the amorphous phase of pure LDPE, EPR data show that an increase in the correlation time of the radical probe from 5.9 × 10^−10^ s in the control to 6.6 × 10^−10^ s after soil exposure indicates a certain increase in rigidity. The similarity of the thermophysical and physicomechanical parameters of the control LDPE samples and the LDPE samples after soil exposure suggests that biodestructive factors do not act on pure polyethylene.

#### 3.2.6. Investigation of the Thermal Stability of the Samples

Investigation of the thermal stability of LDPE and LDPE/NR samples with varying NR content by TGA established the following parameters: the onset temperature of mass loss on the integral curve (T_onset_), the temperatures of 10% (T_10_), 20% (T_20_), 30% (T_30_), 40% (T_40_), and 50% (T_50_) mass loss, as well as the peak on the differential curve, corresponding to the maximum mass loss rate (T_max_).

Table 8 presents the thermogravimetric data for LDPE and LDPE/NR with different NR contents. The data show that the character of thermal degradation of the blends significantly depends on the presence of NR.

The obtained values indicate that LDPE is more resistant to thermal degradation compared to NR, as the values for LDPE are higher than those for NR: *T_onset_* by 32 °C and *T_max_* by 91.5 °C. Interestingly, this difference continues to increase upon the addition of NR to LDPE. For instance, for LDPE/NR 90/10, *T_onset_* increases by 51 °C and *T_max_* by 4 °C compared to pure LDPE. However, a further increase in the NR content in the composition does not lead to an additional enhancement of thermal stability. The *T_onset_* values decrease for the 50/50 composition compared to the 90/10 blend: Δ*T_onset_* = 33 °C, although *T_max_* changes only slightly.

It should be noted that NR can simultaneously play two roles in the blend: stabilizing and promoting thermal degradation, depending on its concentration. The stabilizing factor could be natural stabilizers present in the NR, which diffuse into the LDPE matrix volume. A small amount of NR in an encapsulated state does not contribute significantly to thermal degradation. However, at a content of 50%, natural rubber substantially influences this process. As mentioned above, the two temperature parameters are significantly lower compared to the data for the 90/10 composition. Furthermore, at the 50/50 ratio, the phase structure differs considerably from that of the 90/10 composition. The NR is not encapsulated but forms a phase uniformly distributed throughout the sample volume. Therefore, polyethylene does not hinder the thermal degradation of natural rubber.

It is known that relaxation processes occur in polymers over time. To separate the relaxation processes from the degradation caused by processes in the soil, let us consider the influence of NR on the supramolecular structure of LDPE compositions in the control samples (which were stored in an envelope under standard conditions for 5 years).

Table 9 presents the temperature characteristics of the thermal degradation of the control samples of LDPE and LDPE/NR 90/10 and 50/50, obtained by TGA. Compared to the initial thermal degradation data presented in Table 7, it is evident that 60 months of storage under stationary conditions have practically no effect on the thermal stability of LDPE. The changes do not exceed 2%, which is within the experimental error. A similar pattern is observed for the control samples of LDPE/NR 90/10 and 50/50. *T_onset_* decreases by 10 °C for 90/10 and by 1 °C for 50/50, while *T_max_* remains practically unchanged. This indicates the absence of substantial changes capable of affecting the thermal stability of LDPE. It can be concluded that relaxation processes do occur in the samples but have a minimal influence on the changes in the supramolecular structure, which can be neglected when interpreting the data on the biodegradation of LDPE and LDPE/NR in soil.

When analyzing the role of NR in altering the supramolecular structure of LDPE/NR after 5 years of soil exposure compared to control samples, it should be noted that for pure LDPE, the parameters characterizing the crystalline phase are very similar in both cases. Compared to the initial state, a certain increase in the degree of crystallinity is observed, attributable to the contribution of both physical and chemical relaxation processes.

Table 10 presents the temperature characteristics of the thermal degradation of LDPE and LDPE/NR (90/10 and 50/50) samples after 5 years of soil exposure, obtained by TGA.

The temperature parameters of polyethylene practically coincide with both the control sample data and the initial data. This leads to the conclusion that the thermal stability of LDPE remains unchanged during the 5-year soil exposure.

It is possible that natural stabilizers present in NR, may diffuse into the LDPE matrix volume, as explained earlier, promoting stabilization, which may partially explain the results presented in Table 9. In addtion, biodegradation of LDPE may occurrs in the polymer amorphous regions, which might result in remove some branches in LDPE structure, resulting in an increase in thermal stablility. Nguyen et al. (2024) obtained similar results studing bacterial degradation of LDPE [49].

Regarding the compositions with NR, the thermal stability changed significantly after the five-year period in the soil. This is associated with the absence of NR in the tested samples and the presence of only LDPE, which underwent substantial degradation accompanied by chain scission and the accumulation of oxygen-containing functional groups.

The significant decrease in the onset temperature of mass loss (*T_onset_*)—from 293 °C for the control 90/10 sample to 200 °C for the same composition after soil exposure, and from 271 °C to 230 °C for the 50/50 composition—is apparently related to the presence of low-molecular-weight degraded polyethylene fragments. These fragments undergo thermal degradation first.

The increase in *T_max_* by 3–7 °C indicates that the most stable part of LDPE degrades last. This undoubtedly refers to the best-organized crystallites with the fewest defects.

The obtained data correlate with the results of GPC, DSC, EPR, IR spectroscopy, and microscopy, which demonstrated the formation of defects throughout the entire structure, loss of sample integrity, macromolecular chain scission leading to reduced molecular weight, a decrease in the degree of crystallinity, and changes in the amorphous phase. These factors collectively led to a substantial reduction in the strength characteristics.

#### 3.2.7. GPC

This study obtained key confirmations of biodegradation using GPC for LDPE/NR 90/10 and 50/50 samples. Table 11 presents the molecular weight distribution characteristics for the control samples and the samples retrieved from the soil. Table 10 shows the distribution of macromolecules by molecular weight.

When analyzing the results, it was taken into account that the control samples contain NR as a component, whereas in the samples after soil exposure, NR is completely absent, according to FTIR and TGA data.

For the sample with 10% NR content, a decrease in Mv, Mw, and Mz (viscosity-average, weight-average, and z-average molecular weights, respectively) was detected, primarily contributed to by the high-molecular-weight fraction. Concurrently, an increase in Mp and Mn (relative and number-average molecular weights, respectively) is observed. The proportion of the low-molecular-weight component increases at the expense of larger molecules, which is confirmed by the distribution of macromolecules by molecular weight (Table 12). Based on the obtained data, it can be concluded that the process of polyethylene biodegradation occurs, accompanied by scission of macromolecules in the presence of NR, whereas the molecular weight of LDPE itself remains unchanged.

This study established the role of natural rubber content in the composition. As evident from the GPC analysis of LDPE/NR, all average molecular weight values decrease significantly specifically for the LDPE/NR 50/50 sample. Moreover, Mz, Mw, and Mv decrease to a greater extent compared to Mn. The first three parameters decrease by approximately 70%, while Mn decreases by only 20%. A substantial difference is observed in the molecular weight distribution between the control LDPE/NR 50/50 samples and those aged in soil for 5 years.

This pattern is explained by the fact that chain scission accompanying biodegradation predominantly reduces the proportion of the highest molecular weight chains, whose fragments enrich the low molecular weight fraction (Table 12). For the LDPE/NR 90/10 sample, the sum of all fractions before and after soil exposure is 100%. In contrast, for the control LDPE/NR 50/50 sample, the sum is 100%, whereas for the sample aged in soil for 5 years, this sum significantly deviates from 100%, amounting to 70.7%. It is known that GPC cannot detect oligomeric fragments with molecular weights below 500 g/mol. This indicates the presence of 30% oligomeric fragments by mass in the sample, which initially contained only LDPE macromolecules after soil exposure. This effect is not observed with a low content of biodegradable NR (10%) in the composition. In the 90/10 case, natural rubber forms an inclusion phase, a dispersed phase. This component is encapsulated within the polyethylene matrix, which acts as the dispersion medium.

As shown by the results obtained in this study (water absorption, biodegradation curves, etc.), when the NR content in the composition exceeds 30%, a phase restructuring occurs. Contact is established between previously separated NR domains within the LDPE matrix. By forming a continuous structure, natural rubber becomes more accessible throughout the entire sample volume to bacteria and micromycetes. This leads to a significant acceleration of the biodegradation of the entire sample, including polyethylene. These conclusions agree with the GPC results, where the Mw, Mz, and Mv values for the 50/50 sample are approximately 20% lower than those obtained for the LDPE/NR 90/10 sample.

It is known that during storage under normal conditions with air access, polymers undergo changes under the influence of physical and chemical processes, including oxidative degradation. Natural rubber exhibits higher reactivity in many chemical processes compared to polyethylene, due to the presence of C=C double bonds in its chain, the absence of crystallinity, and the presence of low molecular weight impurities containing various functional groups.

Thus, the combination of two factors—the higher chemical activity of NR and the restructuring of the phase morphology when its content in the samples exceeds 30%—is the key reason for the enhanced degradation of the 50/50 composition compared to the 90/10 sample. Additional evidence supporting the occurrence of oxidative degradation during long-term storage is that the Mn value decreases in the 50/50 sample by only 6% compared to the corresponding value in the 90/10 composition. This means the loss of the low molecular weight polymer fraction is compensated by fragments resulting from the degradation of larger macromolecules.

## 4. Conclusions

In summary, film composite materials based on LDPE and with varying NR contents were produced using the pressing method. An interpenetrating “network-in-network” system with maximum thermodynamic stability was obtained for composites with a rubber content of over 30%. The effect of natural rubber on the polyethylene matrix was determined. The best combination of performance properties was achieved by forming an interpenetrating “network-in-network” structure with 40 and 50% natural rubber, including a 32% increase in surface hydrophilicity and satisfactory physical and mechanical parameters (tensile strength, relative elongation), which allows NR contents of 40 and 50% in LDPE/NR composites to be considered optimal in terms of performance properties. It was found that the largest surface area of the phase boundaries obtained in the sample with 50% natural rubber ensures more efficient water penetration throughout the sample volume. For the first time, a detailed analysis of the effect of natural rubber on the rate and nature of biodegradation of PE/NR composites in soil was conducted using the IR method. The accumulation of protein components on the surface of films with an NR content of over 30% over 24 months was demonstrated, accompanied by the cleavage of the double polyisoprene bond and the formation of oligomers with ketone and aldehyde groups. The dependence of mass loss of samples after aging in laboratory soil for 5 years was determined. The greatest mass loss was observed for the 50/50 LDPE/NR blend. The sample with a 50% natural rubber content lost 70% of its mass over 60 months, while the sample with a 40% NR content lost 38%. Blends with an NR content greater than 30% represent a “network within a network” system with a developed phase boundary. At low NR content, its phase is dispersed within the PE matrix. Large NR particles are encapsulated within the polyethylene matrix, making the rubber poorly accessible. For the first time, a decrease in the molecular weight of polyethylene in PE/NR composites after 5-year storage in soil was observed. A significant drop in all average Mm values occurred. Mz, Mw, and Mv were subject to the greatest decrease compared to Mn. The first three values decreased by approximately 70%, while Mn decreased by only 20%. A significant difference in the molecular weight distribution of 50/50 PE/NR composite samples stored and aged in soil for 5 years is evident.

## Figures and Tables

**Table 1:** The parameters of LDPE.

Parameter	Value
Total number of CH_3_ groups per 1000 carbon atoms	21.6
Number of terminal CH_3_ groups per 1000 carbon atoms	4.5
Ethyl branches	14.4
Total number of double bonds per 1000 carbon atoms, including:	0.4–0.6
vinyl double bonds (R-CH=CH_2_), %	17

**Table 2:** Composition of NR.

Component	%
Rubber	91.0–95.0
Proteins	2.37–3.76
Ash	0.10–0.90
Acetone extract	2.30–3.60
Sugars	0.30–0.35
Water extract	0.20–0.40
Moisture	0.18–0.90

**Table 3:** Sound velocity values.

Sample	Speed of Sound, m/s
LDPE 100	2000 ± 10
LDPE/NR 90/10	1900 ± 10
LDPE/NR 50/50	1470 ± 10

**Table 4:** Characterization of roughness of LDPE/NR systems.

Sample	R_a_, nm ±SD (n = 5)	R_q_, nm ±SD (n = 5)
0	30.52 ± 3	38.25 ± 4
10	18.88 ± 2	22.86 ± 2
50	18.14 ± 2	21.27 ± 2

**Table 5:** Mechanical parameters of LDPE samples.

Parameter	Initial	Control	After Exposure in Soil
Relative elongation, %, (±10%)	625	550	580
Tensile strength, MPa (±0.5 MPa)	15.2	10.4	10.9
Modulus of elasticity, MPa (±0.5 MPa)	87	60	55

**Table 6:** Mechanical parameters of LDPE/NR 90/10 samples.

Parameter	Initial	Control	After Exposure in Soil
Relative elongation, %, (±10%)	120	104	98
Tensile strength, MPa (±0.5 MPa)	6.3	5.8	5.3
Modulus of elasticity, MPa (±0.5 MPa)	62	60	55

**Table 7:** Mechanical parameters of LDPE/NR 50/50 samples.

Parameter	Initial	Control	After Exposure in Soil
Relative elongation, %, (± 10%)	450	250	45
Tensile strength, MPa (±0.5 MPa)	3.9	2.8	4.0
Modulus of elasticity, MPa (±0.5 MPa)	26	23	81

**Table 8:** Temperature characteristics of thermal destruction of the initial LDPE and LDPE/NR samples.

Sample	*T_onset_*, °C	*T*_10_	*T*_20_	*T*_30_	*T*_40_	*T*_50_	*T_max_*, °C
NR	220	363	349	369	378	383	380
LDPE	252	410	435	448	458	465	472
LDPE/NR 90/10	303	417	441	452	460	472	476
LDPE/NR 50/50	270	372	388	387	421	449	476

**Table 9:** Temperature characteristics of thermal degradation for control samples of LDPE and LDPE/NR.

Sample	*T_onset_*, °C	*T*_10_	*T*_20_	*T*_30_	*T*_40_	*T*_50_	*T_max_*, °C
LDPE	252	405	423	441	450	459	467
LDPE/NR 90/10	293	389	429	450	461	473	476
LDPE/NR 50/50	271	371	383	397	418	448	478

**Table 10:** Temperature characteristics of thermal degradation of LDPE and LDPE/NR samples after 5 years of soil exposure.

Sample	*T_onset_*, °C	*T*_10_	*T*_20_	*T*_30_	*T*_40_	*T*_50_	*T_max_*, °C
LDPE	247	399	425	441	450	459	468
LDPE/NR 90/10	200	435	457	465	480	483	482
LDPE/NR 50/50	230	438	460	471	484	487	481

**Table 11:** Results of the GPC analysis of LDPE/NR.

Sample	Mp g/mol	Mn g/mol	Mw g/mol	Mz g/mol	Mv g/mol	PD
LDPE/NR 90/10 control	51.2 × 10^3^	17.0 × 10^3^	142.6 × 10^3^	1162.9 × 10^3^	939.4 × 10^3^	8.37
LDPE/NR 90/10 after exposure	54.4 × 10^3^	17.7 × 10^3^	129.7 × 10^3^	998.4 × 10^3^	802.35 × 10^3^	7.32
LDPE/NR 50/50 control	30.9 × 10^3^	16.0 × 10^3^	108.2 × 10^3^	927.7 × 10^3^	738.6 × 10^3^	6.76
LDPE/NR 50/50 after exposure	20.1 × 10^3^	12.6 × 10^3^	67.8 × 10^3^	267.9 × 10^3^	226.3 × 10^3^	5.37

**Table 12:** Content of fractions in LDPE/NR samples.

	Contents of Factions						Total Amount
Sample	Less 1000 g/mol	1000–10,000 g/mol	10,000–50,000 g/mol	50,000–100,000 g/mol	100,000–1,000,000 g/mol	More 1,000,000 g/mol	PD
LDPE/NR 90/10 control	0.3	14.1	38.1	18.6	26.4	2.5	100
LDPE/NR 90/10 after exposure	0.2	14.1	38.9	19.2	25.6	2.0	100
LDPE/NR 50/50 control	0.3	15.3	40.7	19.9	22.3	1.5	100
LDPE/NR 50/50 after exposure	0.2	13.2	24.1	15.5	16.7	1.0	70.7

## Data Availability

The original contributions presented in this study are included in the article. Further inquiries can be directed to the corresponding author.

## Supplementary Materials

The following supporting information can be downloaded at: https://www.mdpi.com/article/10.3390/polym17212885/s1, Figure S1. TGA curves, where: initial samples (a), control samples (b), samples after exposure in soil (c). Figure S2. DSC curves, where: PE 100 (a), PE/NR 90/10 (b), PE/NR 50/50.
