# Supplementary material for: The Use of Natural Rubber as an Initiator of LDPE Biodegradation in Soil
Source: Polymers (Basel). 2025 Oct 29;17(21):2885. doi: 10.3390/polym17212885 (PMC12610600; doi:10.3390/polym17212885)
Supplement: Supplementary file 1 [file polymers-17-02885-s001.zip › polymers-3947967-supplementary.pdf]

Supplementary Materials

(a)

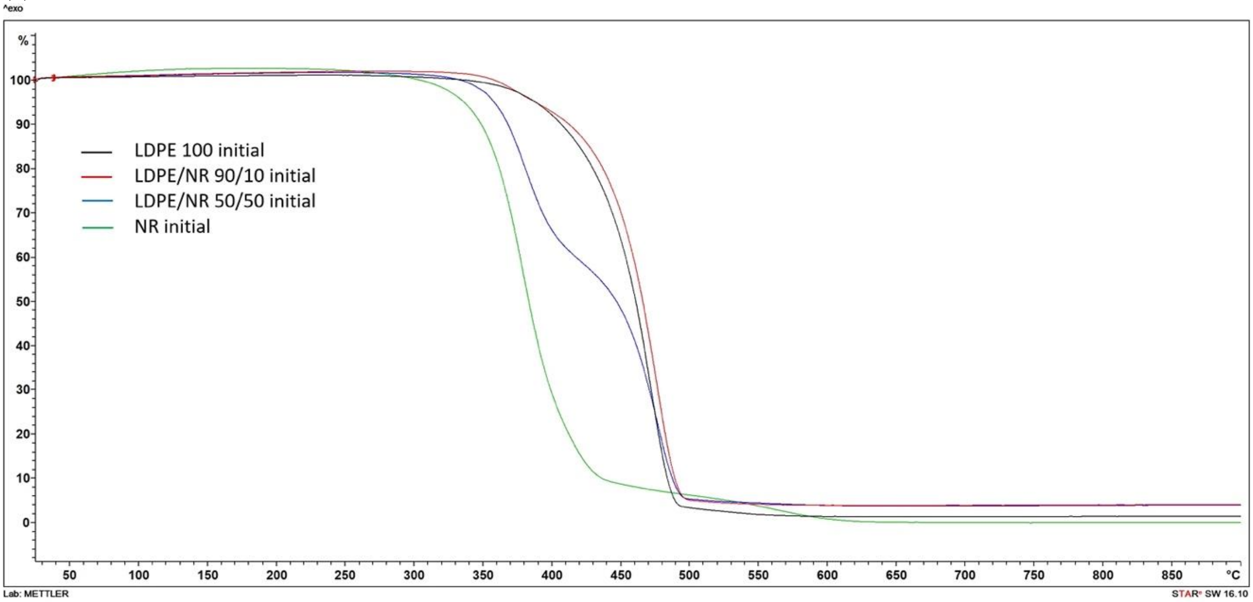

(b)

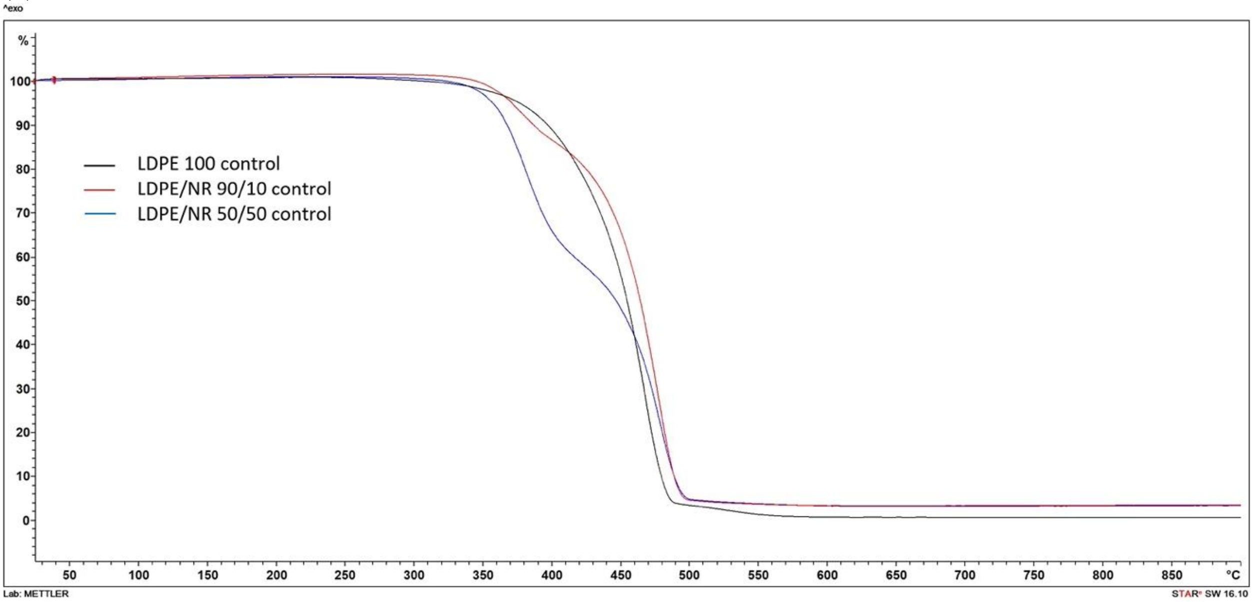

(c)

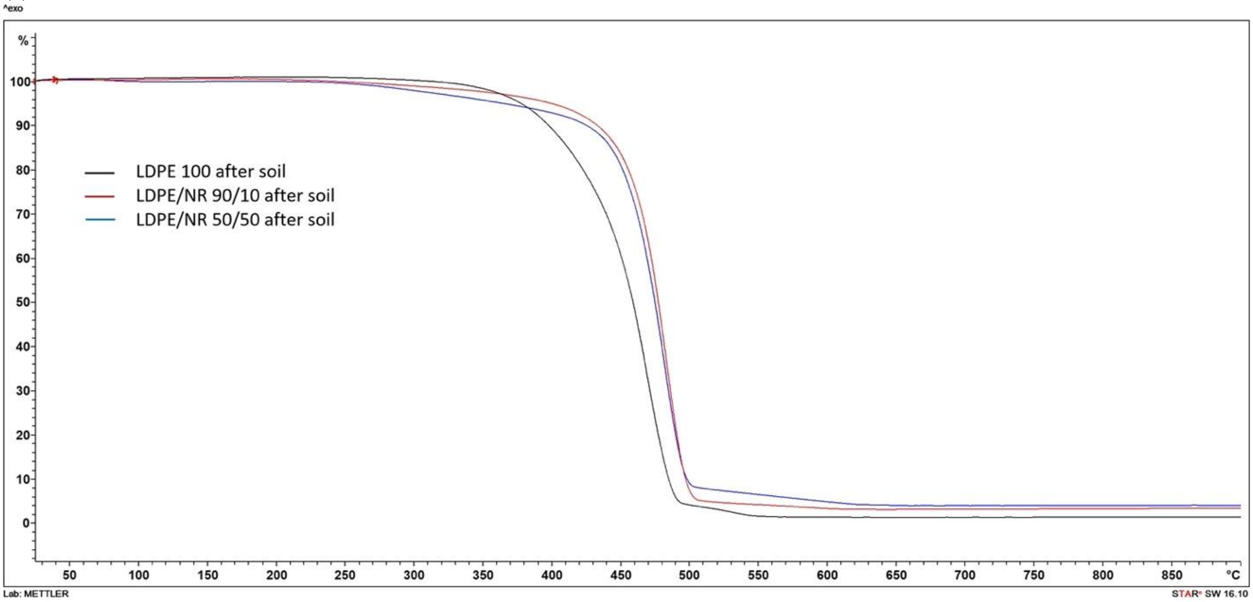

Figure S1. TGA curves, where: initial samples (a), control samples (b), samples after exposure in soil (c).

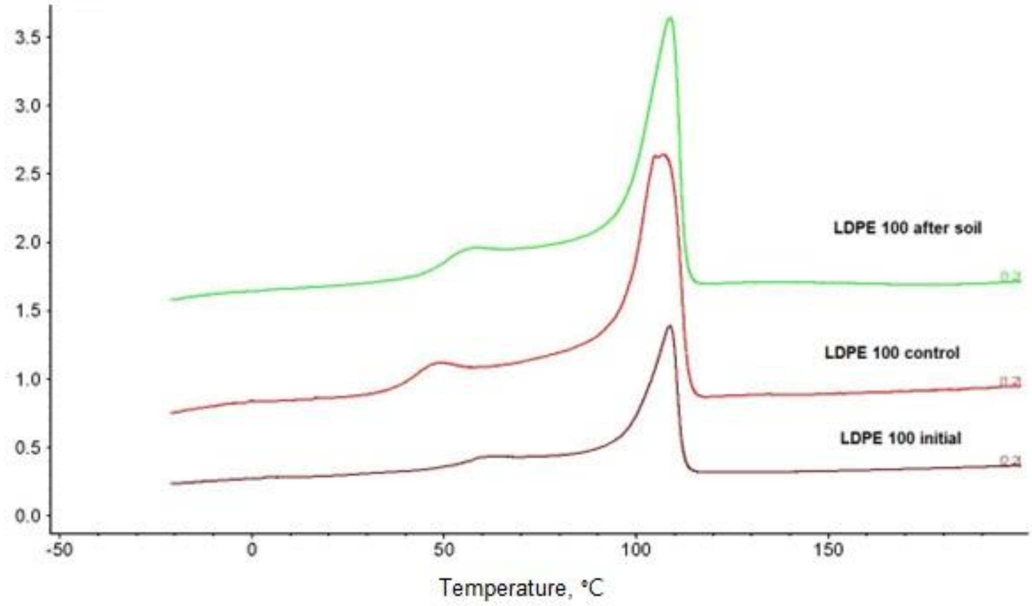

(a)

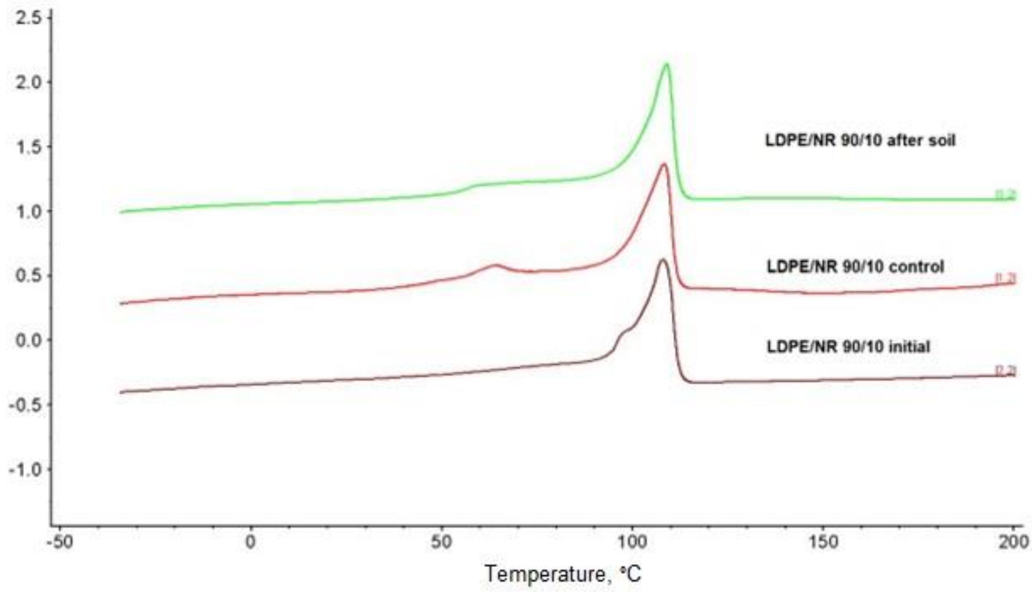

(b)

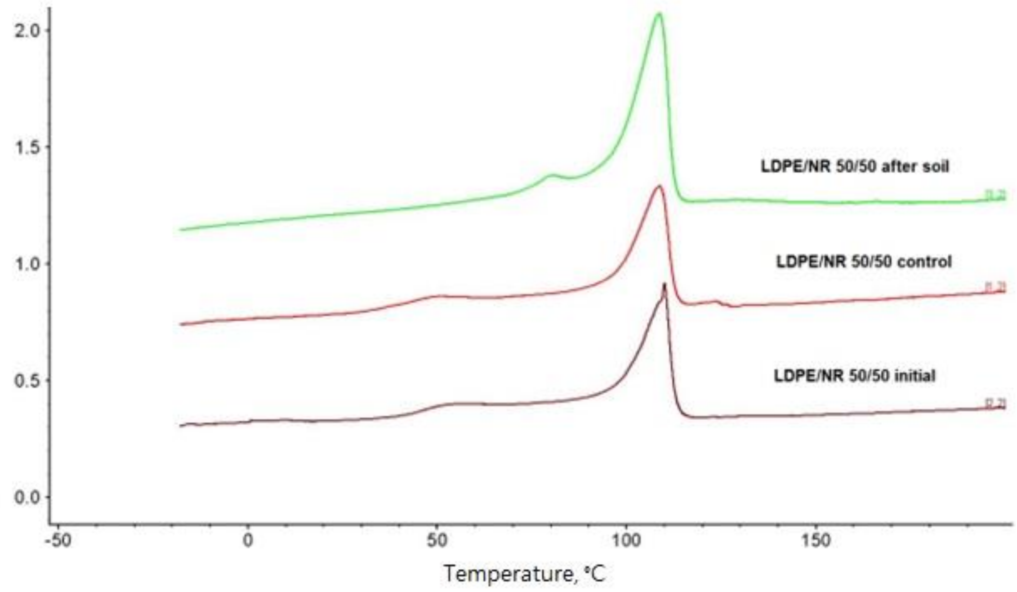

(c)

Figure S2. DSC curves, where: PE 100 (a), PE/NR 90/10 (b), PE/NR 50/50.
